# Form of Service Used at the Unveiling by Her Majesty the Queen of the Memorial to Miss Florence Nightingale, O.M.

**Published:** 1916-02-19

**Authors:** 


### FORM OF SERVICE

USED AT THE UNVEILING BY HER MAJESTY THE QUEEN OF THE

### Memorial to Miss Florence Nightingale, O.M.

St. Paul's Cathedral, February 14, 1916, at 2.15 P.M.

The Queen having unveiled the Memorial in the Crypt at the request of the Archbishop of Canterbury, and having been conducted to a seat under the Dome,

The Priest shall then say—

Let us pray.

Prevent us, O Lord, in all our doings with Thy most gracious favour, and further us with Thy continual help; that in all our works begun, continued, and ended in Thee, we may glorify Thy Holy Name, and finally by Thy mercy obtain everlasting life; through Jesus Christ our Lord. *Amen.*

Our Father, which art in Heaven, Hallowed be Thy Name. Thy kingdom come. Thy will be done in earth, as it is in heaven. Give us this day our daily bread. And forgive us our trespasses, as we forgive them that trespass against us. And lead us not into temptation; but deliver us from evil. *Amen.*

O God, make speed to save us.

*O Lord, make haste to help us.*

Praise ye the Lord.

*The Lord's Name be praised.*

PSALM XXIV.—The earth is the Lord's, and all that therein is.

ANTHEM. *Spohr.*

Blest are the departed, who in the Lord are sleeping, from henceforth for evermore: they rest from their labours and their works follow them.

The Dean shall then say:

These were honoured in their generation and were the glory of their times. There be of them that have left a name behind them, that their praises might be reported; and some there be which have no memorial . . . Their bodies are buried in peace, but their name liveth for evermore.

Let us pray.

We render Thee thanks, O Lord, for the singular gifts which Thou didst bestow upon Thy servant Florence Nightingale, whose Memorial we dedicate in Thy holy house; as well as for all Thy servants which are departed from us in the sign of faith, and now do rest in the sleep of peace; humbly beseeching Thee that it may please Thee of Thy gracious goodness shortly to accomplish the number of Thy elect, that we, with all those that are departed in the true faith of Thy holy Name, may have our perfect consummation and bliss, both in body and soul, in Thy eternal and everlasting glory, through Jesus Christ our Lord. *Amen.*

HYMN No. 428. "That they may rest from their labours."

The Saints of God! their conflict past.

Let us pray.

O Almighty God, Who hast knit together Thine elect in one communion and fellowship in the mystical body of Thy Son Christ our Lord: Grant us grace so to follow Thy blessed Saints in all virtuous and godly living that we may come to those unspeakable joys, which Thou hast prepared for them that unfeignedly love Thee; through Jesus Christ our Lord. *Amen.*

Assist us mercifully, O Lord, in these our supplications and prayers, and dispose the way of Thy servants towards the attainment of everlasting salvation; that, among all the changes and chances of this mortal life, they may ever be defended by Thy most gracious and ready help; through Jesus Christ our Lord. *Amen.*

The grace of our Lord Jesus Christ, and the love of God, and the fellowship of the Holy Ghost, be with us evermore. *Amen.*

